# Penetration of a swallowed fish bone into pulmonary vein: diagnosis and management

**DOI:** 10.1016/j.heliyon.2020.e05611

**Published:** 2020-11-26

**Authors:** Tetsuya Akaishi, Kota Ishizawa, Toshiaki Fukutomi, Yasuchika Yamamoto, Hirofumi Ichikawa, Suguru Watanabe, Naoko Mori, Mayuko Saito, Shin Takayama, Michiaki Abe, Kazuaki Hatsugai, Tadashi Ishii

**Affiliations:** a Department of Education and Support for Regional Medicine, Tohoku University Hospital, Japan; b Department of Surgery, Tohoku University Graduate School of Medicine, Japan; c Department of Gastroenterology, Japanese Red Cross Ishinomaki Hospital, Japan; d Department of Surgery, Japanese Red Cross Ishinomaki Hospital, Japan; e Department of Cardiovascular Surgery, Japanese Red Cross Ishinomaki Hospital, Japan; f Department of Diagnostic Radiology, Tohoku University Graduate School of Medicine, Japan; g Minamisanriku Hospital, Japan

**Keywords:** Medical imaging, Gastrointestinal system, Emergency medicine, Internal medicine, Radiology, Chest computed tomography, Endoscopic removal, Mediastinitis, Penetration, Pulmonary vein, Swallowed fish bone

## Abstract

We present a case of a 71-year-old woman who accidently swallowed a large fish bone that penetrated into the pulmonary vein. She visited the hospital the next day with a complaint of mild chest discomfort with slight pain and fever of 37.4 °C. Contrast-enhanced chest computed tomography (CT) scan revealed a large fish bone with a length of 35 mm impacted in the middle esophagus. The bone had penetrated into the pulmonary vein, causing mediastinitis. Blood tests revealed elevation in the white blood cell count and C-reactive protein level. Because intractable bleeding from pulmonary vein after endoscopic removal can be lethal, endoscopic removal of the fish bone in an operating room under general anesthesia with cardiovascular surgical standby for possible emergency surgery was selected. After endoscopic removal, mediastinal hematoma was absent with a follow-up chest CT scan, and the mediastinitis was treated with intravenous antibiotics. The patient shortly became afebrile with normalized blood test findings. After confirming the normal findings on the follow-up chest CT scan and endoscopic inspection in the next week, she was discharged from the hospital 10 days after hospitalization without any complications. When the swallowed bone penetrates into the major pericardial vessels, unprepared endoscopic removal may result in fatal sequelae such as intractable mediastinal hemorrhage. Urgent consultation with cardiovascular or thoracic surgeons for a possible emergent surgery is needed before endoscopic removal is attempted.

## 1 Introduction

A swallowed foreign body such as a fish bone usually passes through the esophagus spontaneously without the requirement of therapeutic removal [1]. However, an impacted intra-esophageal fish bone could result in severe and fatal consequences such as penetration or perforation without proper diagnosis followed by appropriate therapeutic interventions. We report a very rare case of an impacted esophageal large fish bone that penetrated into the pulmonary vein, causing mediastinitis. It was removed under general anesthesia in the operating room with a cardiovascular surgical standby.

## 2 Case presentation

**Figure 1:**
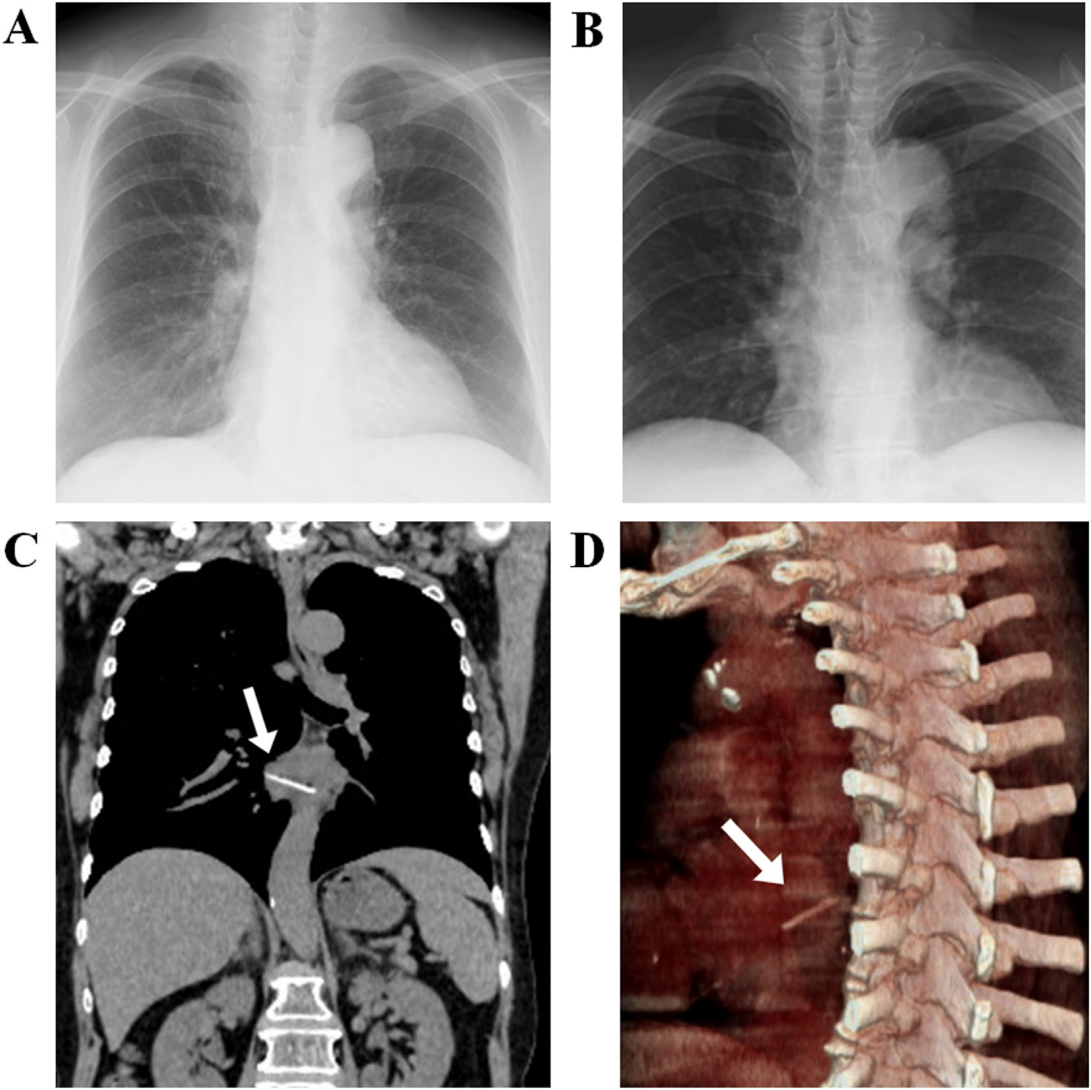
Chest X-ray and non-contrast chest computed tomography. (A, B) Chest X-ray failed to detect any abnormal findings, including the supposedly lodged fish bone in the esophagus. (C, D) Non-contrasted chest CT revealed a large fish bone impacted in the middle esophagus, penetrating the esophageal wall into the surrounding mediastinal structures.

**Figure 2:**
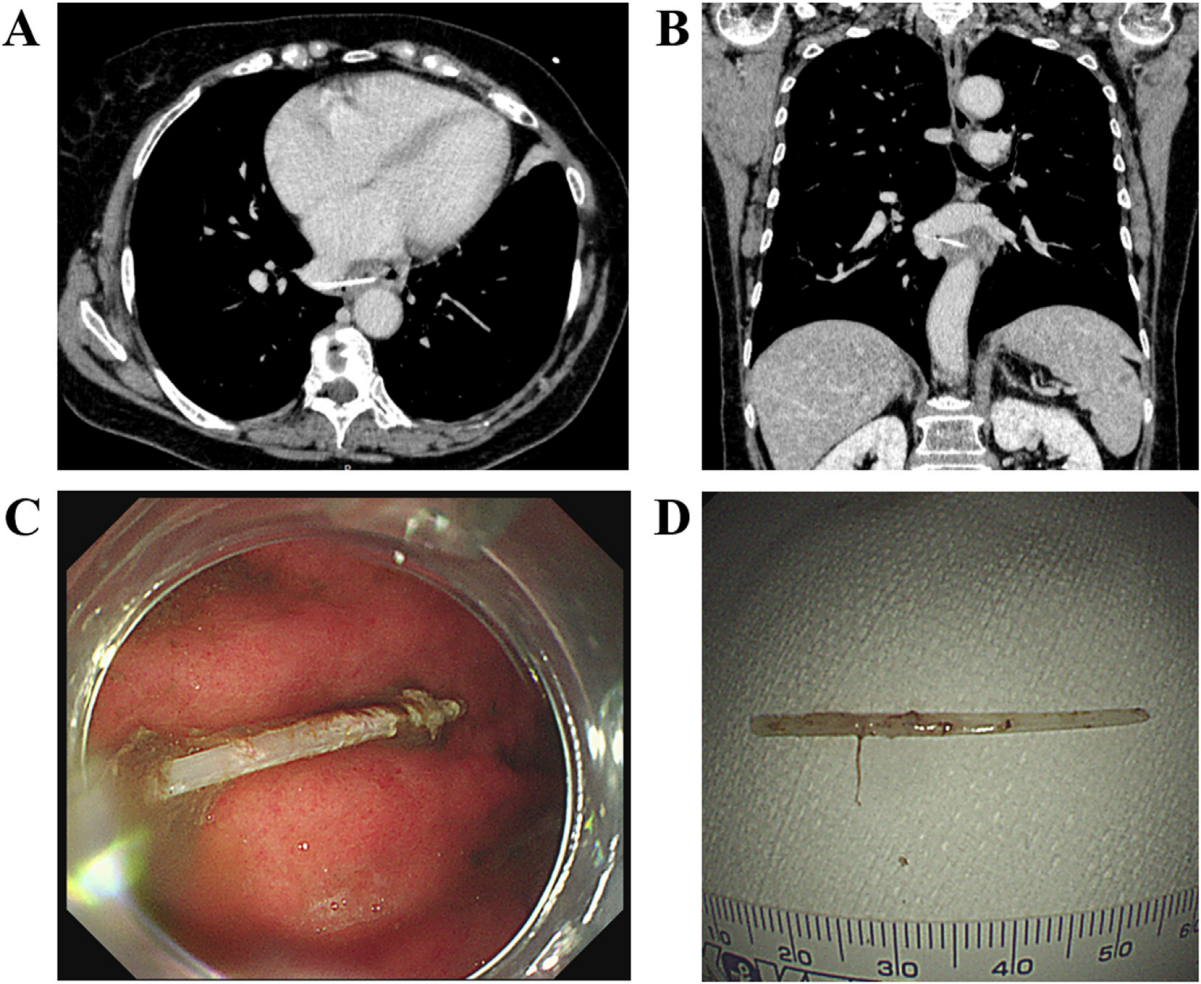
Contrast-enhanced chest computed tomography images and endoscopic findings on bone removal. The axial view (A) and the coronal view (B) of the chest computed tomography scan with a slice thickness of 1 mm revealed a fish bone of 35 mm in length. It penetrated from the right side of the middle intrathoracic esophagus into the right inferior pulmonary vein, accompanied by a small amount of air bubbles. Slice thickness: 1 mm, window level: 50 HU, window width: 300 HU. (C, D) Endoscopic finding on the bone removal, showing the impacted bone with 35 mm length.

A 71-year-old woman with an unremarkable medical history visited our hospital after she accidently swallowed a tuna bone at supper. She complained of mild chest discomfort with slight pain. Chest radiography revealed no abnormal findings and she was permitted to go home at night (Figure 1A, B). The next morning, she revisited the hospital with deterioration of chest discomfort and a slight fever of 37.4 °C. Non-contrast chest computed tomography (CT) revealed an impacted large fish bone with a length of 35 mm lying across the middle third of the thoracic esophagus and accompanied by local mediastinitis (Figure 1C, D). She was immediately referred to a larger hospital, where she underwent contrast-enhanced chest CT scan that revealed penetration into the right inferior pulmonary vein caused by the fish bone (Figure 2A, B). The bone protruded more than 10 mm from the vessel wall inside the pulmonary vein, accompanied by a small amount of free air. Blood tests revealed an abnormally elevated white blood cell (WBC) count of 11,400/μL and elevated C-reactive protein (CRP) level of 20.45 mg/dL. Although the pulmonary vein is a low-pressure system, endoscopic removal may result in a lethal intractable bleeding that would require an emergent cardiovascular surgery. Thus, endoscopic removal of the fish bone was performed in the operating room under general anesthesia (Figure 2C, D) with cardiovascular surgical standby for a possible urgent surgery with a right thoracotomy approach.

The impacted fish bone was successfully removed endoscopically without massive bleeding. A follow-up chest CT scan was performed just after the endoscopic removal that confirmed the absence of mediastinal hematoma. The patient was carefully managed in the emergency ward not to overlook possible sudden changes of the general condition. Oral feeding was prohibited until the follow-up of chest CT scan and endoscopic inspection planned in the next week. To treat the mediastinitis, 3 g/day of intravenous meropenem was initiated. Fever, WBC count, and CRP level were normalized by day 7 after the endoscopic removal. Contrast-enhanced chest CT scan and endoscopic follow-up at 8 days after the removal confirmed resolution of mediastinitis and the absence of abscess or hematoma, after which the patient was permitted to start oral feeding. The patient was discharged from the hospital at 10 days after hospitalization without any major complications.

### 2.1 Declaration of patient consent

Written informed consent was obtained from the patient.

## 3 Discussion

Swallowed fish bone is one of the most frequent intra-esophageal foreign bodies in adults. The compaction of the fish bone usually takes place at the physiological stricture of the esophagus [2]. As a result, penetration of a fish bone into major pericardial vessels like the present case is a very rare condition. Laryngoscopy or upper endoscopy is the most reliable diagnostic method with the highest sensitivity. Moreover, clinicians can simultaneously remove the fish bone using this diagnostic method. However, endoscopic examinations are invasive and not all clinicians can perform the maneuver. Chest CT is another useful diagnostic examination, especially when acquired in thin slices. Chest radiography is not an appropriate method for screening an intra-esophageal object, as it has a sensitivity of approximately 50% for detecting impacted fish bones. In contrast, CT scans using a slice thickness of 3 mm or less can detect almost all large-sized fish bone impactions [3]. It should be noted that patients with swallowed fish bone may sometimes complain of chest discomfort in an area different from the exact location of impaction, especially when the impaction site is in the lower esophagus [2, 4]. In addition, patients with impacted fish bone in the esophagus often complain only of slight to mild chest symptoms on their first visit. Consequently, a chest CT scan is recommended as a noninvasive and reliable diagnostic tool in cases with swallowed foreign bodies causing chest discomfort. Ideally, contrast-enhanced chest CT scans should be performed for all cases with impacted intra-esophageal large fish bones before endoscopic removal, as the penetrated fish bone may damage the surrounding major pericardial vascular structures. Massive bleeding or pneumothorax is a conceivable serious sequela after endoscopic removal. If the bone penetrates into major pericardial vessels, consultation with cardiovascular surgeons is needed before endoscopic bone removal is attempted. Particularly, when the fish bone penetrates into the aorta (i.e., a high-pressure system), cardiovascular surgery with thoracotomy would be better than unprepared endoscopic bone removal. A swallowed bone that passes through the esophagogastric junction does not usually require further treatment. However, it could cause perforation in the small intestine in rare instances, which may require an emergent surgery [5, 6]. In such cases, an abdominal CT scan would be essential for the correct diagnosis.

This case suggests the importance of performing contrast-enhanced chest CT scan in cases with an esophageal large fish bone. When the bone penetrates into the major pericardial vessels, unprepared endoscopic removal may result in fatal sequelae such as intractable mediastinal hemorrhage. Urgent consultation with cardiovascular or thoracic surgeons is necessary before the endoscopic removal is attempted.

## Declarations

### Author contribution statement

All authors listed have significantly contributed to the investigation, development and writing of this article.

### Funding statement

This research did not receive any specific grant from funding agencies in the public, commercial, or not-for-profit sectors.

### Declaration of interests statement

The authors declare no conflict of interest.

### Additional information

No additional information is available for this paper.
